# Insight into Gene Polymorphisms Involved in Toll-Like Receptor/Interferon Signalling Pathways for Systemic Lupus Erythematosus in South East Asia

**DOI:** 10.1155/2014/529167

**Published:** 2014-02-17

**Authors:** Hwa Chia Chai, Kek Heng Chua, Soo Kun Lim, Maude Elvira Phipps

**Affiliations:** ^1^Jeffrey Cheah School of Medicine and Health Sciences, Monash University, Sunway Campus, 46150 Selangor, Malaysia; ^2^Department of Biomedical Science, Faculty of Medicine, University of Malaya, 50603 Kuala Lumpur, Malaysia; ^3^Department of Medicine, Faculty of Medicine, University of Malaya, 50603 Kuala Lumpur, Malaysia

## Abstract

Polymorphisms in genes involved in toll-like receptor/interferon signalling pathways have been reported previously to be associated with SLE in many populations. This study aimed to investigate the role of seven single nucleotide polymorphisms within *TNFAIP3*, *STAT4,* and *IRF5*, which are involved in upstream and downstream pathways of type I interferon production, in SLE in the South East Asian populations. Genotyping of 360 Malaysian SLE patients and 430 normal healthy individuals revealed that minor alleles of *STAT4* rs7574865 and rs10168266 were associated with elevated risk of SLE in the Chinese and Malay patients, respectively (*P* = 0.028, odds ratio (OR) = 1.42; *P* = 0.035, OR = 1.80, respectively). Polymorphisms in *TNFAIP3* and *IRF5* did not show significant associations with SLE in any of the ethnicities. Combined analysis of the Malays, Chinese, and Indians for each SNP indicated that *STAT4* rs10168266 was significantly associated with the Malaysian SLE as a whole (*P* = 0.014; OR = 1.435). The meta-analysis of *STAT4* rs10168266, which combined the data of other studies and this study, further confirmed its importance as the risk factor for SLE by having pooled OR of 1.559 and *P* value of <0.001.

## 1. Introduction

Systemic lupus erythematosus (SLE) is a prototypic autoimmune disease affecting various parts of the body including skin, kidneys, lungs, joints, heart, nervous system, and hematopoietic organs. It is a disease whereby a diverse array of autoantibody production, complement activation, immune complex deposition, and inflammation cause damages in those organs. Although the exact aetiology of SLE still remains unclear, a combination of genetic risk factors and environmental events is believed to contribute to an irreversible break in immunological self-tolerance. With the introduction of genome-wide association studies, a huge breakthrough has been made in the discovery of SLE associated susceptibility genes that in turn advances our understanding of pathogenesis of SLE. Recently, several reviews have categorised the susceptible genes according to their immunological pathways and cell types. Three biological pathways involved in SLE have been forwarded by Harley et al. [1]: (i) innate immune response including toll-like receptor (TLR)/interferon (IFN) signalling pathways; (ii) adaptive immune response including B, T, and antigen-presenting cells immune signal transduction; and (iii) immune complex clearance mechanism.

Defects in TLR/IFN signalling pathways cause immune complexes containing self-nucleic acids to interact with TLR7 and TLR9 inside plasmacytoid dendritic cells and B cells endosomes, resulting in the secretion of type I IFN and interleukin (IL)-6. The combined triggering of both B cell receptors and TLR leads to autoreactive B-cell proliferation. Their further differentiation into plasmablasts and autoantibody-secreting plasma cells is induced by type I IFN and IL-6, respectively [2]. *TNFAIP3*, *STAT4*, *IRF5*, *TREX*, and *IRAK1 *are the genes involved in upstream and downstream pathways of type I IFN production that have been recently identified. The *STAT4 *gene consists of 24 exons that spread over a 120 kb region on chromosome 2q32.3. It encodes a transcription factor that mediates signals induced by IL-12, IL-23, and type I IFN and activates the production of IFN-*γ* and IL-17. It also directs the differentiation of helper T cells toward the proinflammatory T-helper type 1 and T-helper type 17 lineages that have been shown to play a critical role in the pathogenesis of SLE. The *STAT4* null allele in lupus-prone mouse model confers reduced autoantibody production and glomerulonephritis, indicating that *STAT4* may be involved in multiple SLE-associated phenotypes [3]. There are a few studies involving *STAT4*-deficient lupus-prone mice which demonstrate the role of *STAT4* in autoantibody production only [4, 5]. Polymorphisms in the *STAT4 *gene have been found to be strongly associated with SLE susceptibility, in particular rs7574865 [6, 7]. The simultaneous association of the risk allele T of *STAT4 *rs7574865 with both lower serum IFN-*α* activity and increased IFN-*α*-induced gene expression has been reported, confirming that this polymorphism was associated with increased IFN-*α* sensitivity [8, 9].

*TNFAIP3*, or tumour necrosis factor alpha-induced protein 3 gene, encodes the A20 protein which is a negative regulator of the NF-*κ*B signalling pathway, an essential pathway in the pathogenesis of SLE. A20 is an ubiquitin-editing enzyme required for effective termination of NF-*κ*B-mediated proinflammatory responses induced by TLRs, TNF receptor, IL-1 receptor, and NOD2 [10]. A meta-analysis and imputation study identified a 109 kb risk haplotype spanning *TNFAIP3 *region with lupus nephritis and hematologic manifestation [11]. A nonsynonymous mutation (c.380T > G), rs223092, in *TNFAIP3* gene which causes phenylalanine-to-cysteine change at position 127 of A20 protein has been consistently linked with SLE various ethnic groups.

The final candidate gene, *IRF5*, which is IFN regulatory factor 5, is a transcription factor that mediates inflammatory and immune responses [12]. This factor stimulates the production of the proinflammatory cytokines TNF-*α*, IL-12, and IL-6 following TLR signalling as well as transactivation of type I IFN and IFN-induced genes [13, 14]. Polymorphisms in *IRF5* cause functional changes in messenger RNA, which in turn alter *IFR5*-mediated transcription resulting in elevated SLE risk [15]. It was also suggested that SLE patients who carry *IRF5 *risk haplotype and are positive for either anti-RBP or anti-dsDNA potentially have higher serum IFN-*α* activity [16]. In this study, we aimed to investigate the association between seven single nucleotide polymorphisms (SNPs) in *STAT4*, *TNFAIP3*, *IRF5* genes, and SLE in the South East Asian scenario, particularly in the Malaysian participants. We also attempted to compare and pool the ORs of SNPs which were significant in the Malaysian SLE with the other studies through meta-analysis.

## 2. Materials and Methods

### 2.1. Sample Collection and DNA Extraction

A total of 790 Malaysians were included in this study, which is comprised of 360 SLE patients and 430 healthy controls. Blood samples were collected from patients diagnosed with SLE according to 4 out of ACR criteria and healthy volunteers recruited at the University of Malaya Medical Centre (UMMC), Kuala Lumpur, in compliance with requirements as stipulated by the UMMC Medical Ethics Committee (UMMC Ethics Approval Code: 733.19). The distribution of samples from Malays, Chinese, and Indians, as well as the ratio of females to males, is shown in Table 1. Genomic DNA was isolated from the peripheral blood samples by using the standard DNA extraction method as described previously [17]. The concentration and purity of the extracted DNA were further quantified by measuring the absorbance values at 260 nm and 280 nm via a spectrophotometer.

### 2.2. Genotyping with Tetraprimer ARMS-PCR

SNPs that were included in this study are listed in Table 2. Tetraprimer ARMS-PCR was performed in the genotyping of rs10168266 and rs7601754 in *STAT4* region, rs2230926 and rs3757173 in *TNFAIP3 *region, and rs4728142 in *IRF5* region. Primers were designed using computer software accessible through the Internet at http://cedar.genetics.soton.ac.uk/public_html/primer1.html, developed by Ye and team [18]. *In silico* PCR as described previously was further carried out to ensure the self-designed primers were targeted to the gene regions of interest [19–21]. Each PCR reaction was carried out in a total of 10 *μ*L, containing 50 ng of template DNA, appropriate concentration of inner and outer primers and MgCl_2_ (Table 3), 200 *μ*M dNTP, 20 mM Tris-HCl pH8.4, 50 mM KCl, and 0.15 U *Taq *polymerase (Fermentas, Vilnius, Lithuania.). The PCR mixture was then subjected to touchdown PCR, whereby it was incubated for 5 min at 95°C, followed by 30 cycles of 45 s denaturation at 95°C, 45 s of annealing (started at temperature 10°C higher than annealing temperature, decreasing by 1°C per cycle, maintained at annealing temperature for the remaining 20 cycles) and 45 s of extension at 72°C, and a final extension at 72°C for 10 min at the end of the cycles. The annealing temperatures for different PCRs are stated in Table 3. Five microlitres of PCR amplicons was electrophoresed on a 2% (w/v) agarose gel. The agarose gel was viewed under UV illumination and image was recorded using a gel documentation system. The results obtained were further verified by sequencing.

### 2.3. Genotyping with Real-Time PCR

Predesigned TaqMan SNP genotyping assays were used to genotype SNPs where tetraprimers could not be designed for ARMS-PCR (probe ID: rs7574865 in *STAT4* region, C_29882391_10; rs729302 in *IRF5* region, C_2691216_10; Applied Biosystems, NY, USA). Fifty nanograms of template DNA was mixed with 2X Taqman GTXpress master mix (Applied Biosystems) and 20X Taqman genotyping assay (Applied Biosystems) to make up to a total volume of 10 *μ*L. Real-time PCR reaction was initiated with pre-PCR read step at 60°C for 1 min, followed by DNA polymerase activation at 95°C for 20 s, 40 cycles of denaturation (95°C for 3 s) and annealing/extension (60°C for 30 s), and ended with a final extension step at 60°C for 1 min. Fluorescence was detected using an Applied Biosystems 7500 Fast Real-Time PCR System. The results were verified by sequencing.

### 2.4. Association Test

Allele and genotype frequencies were calculated, followed by performing a *χ* ^2^ goodness-of-fit test to evaluate whether or not the observed genotype frequencies of each polymorphisms were departures from Hardy-Weinberg equilibrium (HWE) in control subjects (*P*  values > 0.05). An open access HWE calculator developed by Rodriguez et al. [22] was used. Fisher's exact test was conducted on 2 × 2 contingency table using SPSS software to assess the association of each SNP with SLE susceptibility in Malays, Chinese, and Indians. *P* values were adjusted according to Bonferroni correction and *P* < 0.05 was regarded as significant. Odds ratios (ORs) with 95% confidence intervals (CIs) were calculated. Adjusted ORs were computed using logistic regression, whereby major allele and major homozygous genotype of each SNP were set as reference group and their ORs were adjusted to 1.

The ORs of the three ethnicities were combined using Mantel-Haenszel test to evaluate the overall association of each SNP in the Malaysian population. The Mantel-Haenszel ORs were calculated using Comprehensive Meta-Analysis Version 2.0 software (Biostat, NJ, USA). The between-subgroup heterogeneity was tested using Cochran's Q statistic. Random effect model was used when heterogeneity was significant (*P* < 0.10); otherwise, the fixed effect model was used.

### 2.5. Meta-Analysis

Meta-analysis was conducted using Comprehensive Meta-Analysis Version 2.0 software (Biostat) for the SNP(s) which was/were significantly associated with SLE in the Malaysian population by including data from other studies as well as the current study. Due to insufficient number of Indian subjects, analysis was not carried out for this ethnic group.

We examined the association between *STAT4* polymorphisms and SLE fully and rigorously, with the use of the key words “*STAT4*,” “polymorphisms,” “systemic lupus erythematosus,” and “SLE.” Electronic databases including Pubmed, Embase, and Web of Science were thoroughly searched until December 2013. Only fully published articles were included and the eligible studies were identified based on the following criteria: (a) the study was original, (b) the patients were sporadic cases, (c) having available allele and genotype frequency data, and (d) having sufficient published data to determine OR with 95% CI.

Data extraction was performed by collecting the following information from each study: the first author's name, year of publication, ethnicity, the number of cases and controls, and the frequency of minor allele (MAF) of each polymorphism in both cases and controls. For studies including several independent case-control populations, each case-control population was extracted separately. Malay and Chinese populations from this study were also included in the meta-analysis.

The heterogeneity across studies was evaluated by using Cochran's Q statistic. Random effect model was used for meta-analysis when heterogeneity was significant (*P* < 0.10); otherwise, fixed effect model was used. By inputting the study name, total number of cases and controls, and MAF of cases and controls, Comprehensive Meta-Analysis Version 2.0 software calculated the ORs, 95% CI, and *P* values for each study, as well as the pooled OR, 95% CI, and *P* values for the meta-analysis.

## 3. Results

### 3.1. Polymorphisms and SLE Risk

The *χ* ^2^ goodness-of-fit test demonstrated that all polymorphisms investigated in this study fulfilled HWE in the control group. The Malay SLE patients were significantly associated with minor alleles of *STAT4* rs7574865, rs10168266, and *TNFAIP3* rs2230926, and heterozygous genotype TG of *TNFAIP3* rs2230926 (Table 4). Increased SLE susceptibility in Chinese population was significantly conferred by minor alleles and minor homozygous genotypes TT of *STAT4* rs7574865 and rs10168266. However, after Bonferroni adjustment, significant associations were only observed between minor allele T of *STAT4 *rs7574865 and Chinese SLE patients (*P* = 0.028, OR = 1.42, 95% CI: 1.12–1.82) and between minor allele T of *STAT4* rs10168266 and Malay SLE patients (*P* = 0.035, OR = 1.80, 95% CI: 1.20–2.71). *STAT4 *rs7601754, *TNFAIP3* rs3757173, and *IRF5* rs4728142 and rs729302 did not show significant association with SLE in any of the ethnicities.

Combined analysis of the three ethnicities was carried out to represent the association of each SNP with SLE in the Malaysian population as a whole. The analysis revealed that only minor allele T of *STAT4* rs10168266 was significantly associated with the Malaysian SLE (*P* = 0.014, OR = 1.435, 95% CI: 1.143–1.802) (Table 5).

### 3.2. Meta-Analysis of STAT4 rs10168166

Since *STAT4 *rs10168166 showed significant association with the Malaysian SLE, meta-analysis was carried out to combine the data from other studies with the current one [6, 23–25]. Four relevant articles were identified eligible and a total of 5 subgroups were included for comparison. Four subgroups were from Asian population, while one was from European population. Data extracted from these articles is shown in Table 6.

**Figure 1:**
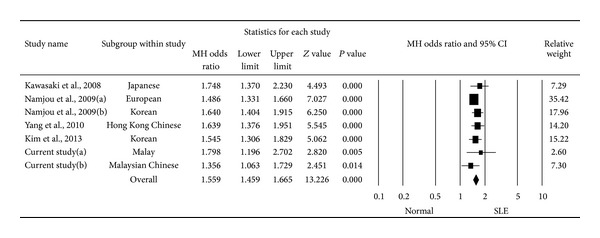
Forest plot of individual and pooled ORs with 95% CI for *STAT4 *rs10168266 with SLE risk.

In the overall analysis, significant association of *STAT4 *rs10168166 with SLE was observed. The fixed effect model was used as the heterogeneity test did not appear significant (*P* > 0.10). The pooled OR for the minor allele T was 1.559, with 95% CI of 1.459–1.665 and *P* value of <0.001 (Figure 1).

## 4. Discussion

Understanding the full molecular pathology of SLE remains a great challenge, although many insights have been revealed. Recognition of self-nucleic acids by toll-like receptors TLR7 and TLR9 on plasmacytoid dendritic cells and B cells is believed to be an important step in the pathogenesis of this disease [26]. Increased antinuclear antibodies and production of type I IFN are both correlated with the severity of disease. *STAT4*, *TNFAIP3*, and *IRF5* are genes involved in regulating TLR/IFN signalling pathways. SNPs investigated in this study have consistently shown associations with SLE susceptibility in many populations, especially in Asians [7, 24, 27–29]. When the various ethnic groups were considered, rs7574865 and rs10168266 of *STAT4* gene were significant in Chinese and Malays, respectively. However, only rs10168266 of *STAT4 *was observed to have correlations with SLE in the Malaysians generally. None of the SNPs seemed to influence SLE in Indians. Due to population demographics and lower SLE risk predisposition, the fewer Indians recruited in this study may have impacted the results. The SNPs of *IRF5* were not significant. This suggests that the *IRF5* genetic variants tested for in this study are not linked to SLE in our cohort and that there may be other variants that are more important in the Indian ethnic group.

Therefore, it may be concluded from the present study that *STAT4* gene polymorphisms feature more prominently as the genetic risk factors in the Malaysian SLE rather than those polymorphisms in *TNFAIP3 *and *IRF5*. Rs10168266 which is located in intron 5 of *STAT4 *gene has been frequently related to SLE susceptibility in the Asian population, particularly in Korean population, and also in the European population [6, 23–25, 30]. This was also reflected in the findings of this study. Nevertheless, not many studies were done on this SNP and thus only four studies were included in the meta-analysis of this study. After the analysis, the pooled OR and *P* value once again showed that this SNP was overall an important risk factor for SLE and more attention should be taken.

Rs7574865, which was significantly associated with SLE in Chinese in this study, is located in the third intron of the *STAT4* gene. The minor/risk allele T has reported associations with other immune-mediated diseases such as rheumatoid arthritis, primary Sjögren's syndrome, type-1 diabetes, Crohn's disease, and ulcerative colitis [31–34]. The association of this particular SNP with SLE susceptibility was observed in many populations, including both European and Asian populations [7]. SNP haplotype in the third intron of *STAT4* marked by rs7574865 was found to be associated with SLE susceptibility and it could be responsible for splice variation or regulatory effects of *STAT4 *[31, 35].

The next SNP that may be important in the Malaysian SLE was *TNFAIP3 *rs2230936. This coding SNP is a nonsynonymous variant causing a phenylalanine-to-cysteine change at residue 127 of the A20 protein. It has been already proven that minor Cys127 is relatively stable compared to the Phe127 protein, causing it to be less effective at inhibiting TNF-induced NF-*κ*B activity [36]. This reduced autoinflammatory activity of A20 could result in excessive cellular response to TNF. Interestingly, as opposed to other findings suggesting that minor allele G was the risk factor of SLE, the results of this study demonstrated that it conferred protection against SLE in our cohort [11, 24, 27, 36–40]. This study speculates that apart from rs223093, other factors such as adjacent SNPs may possibly alter the structure of A20 protein. This may play a role in SLE susceptibility in the Malays. We suggest that multiple (at least four) genes may collectively play critical roles in the development of this disease [41–43]. This speculation has yet to be validated.

Finally, both SNPs in *IRF5* gene investigated in our study were not significant in the Malaysian patients although both are fairly established SLE risk factors for Europeans and some Asians [44–47]. Presumably, other SNPs of this gene would have to be considered.

## 5. Conclusion

The present study was relatively small in contrast to larger studies of SLE by other researchers. Nevertheless, we present evidence to suggest that the genes involved in TLR/IFN signalling pathways, especially *STAT4* rs10168266 polymorphisms, contribute to the development of SLE in Malays and Chinese.

## Figures and Tables

**Table 1:** Distribution of samples according to ethnicity and gender. The percentage of SLE patients and healthy controls was ethnic- and gender-matched.

	SLE patients	Healthy controls
Total	360	430
Malay	93 (25.8%)	110 (25.6%)
Chinese	245 (68.1%)	294 (68.4%)
Indian	22 (6.1%)	26 (6.0%)
Female : Male	10.25 : 1	10.03 : 1

**Table 2:** SNPs that were investigated in this study on their association with SLE.

Genes	SNP	Chromosome	Position	Alleles
*STAT4 *	rs7574865	2	191672878	G/T
	rs10168266	2	191644049	C/T
	rs7601754	2	191648696	A/G

*TNFAIP3 *	rs2230936	6	138237759	T/G
	rs3757173	6	138231847	T/C

*IRF5 *	rs4728142	7	128361203	G/A
	rs729302	7	128356196	A/C

**Table 3:** Touchdown PCR primers and conditions.

SNP	Primer sequence	*T*_*m*_	Final concentration	Inner/outer primers ratio	Mg^2+^	Annealing temperature
*STAT4 *						
rs10168266	Forward inner primer (T allele) (29 bp) 5′-CAAAGTAGTAGCTATTGACTACATGAGAT	57°C	1.0 *μ*M	4 : 1	2.5 mM	55°C
	Reverse inner primer (C allele) (27 bp) 5′-GTTATTACTACGGGTGGGTAGACATTG	62°C	1.0 *μ*M			
	Forward outer primer (28 bp) 5′-AAAAGTATAGAATTTGGAGGAAGAGAGT	59°C	0.25 *μ*M			
	Reverse outer primer (28 bp) 5′-TATTGGGGTATACTGAAAAGAAAGAGTA	59°C	0.25 *μ*M			
rs7601754	Forward inner primer (A allele) (21 bp) 5′-GGGTGAAGAAAAGGAACTCCA	60°C	1.0 *μ*M	5 : 1	1.25 mM	55°C
	Reverse inner primer (G allele) (23 bp) 5′-CAAGGTCTTAGTATCATCTTGGC	57°C	1.0 *μ*M			
	Forward outer primer (28 bp) 5′-GGAGGTGATTACTATATTTCTAGGCTAA	58°C	0.2 *μ*M			
	Reverse outer primer (27 bp) 5′-AAAAATTAAAAATTAGTTGGCTATGGT	58°C	0.2 *μ*M			

*TNFAIP3 *						
rs2230936	Forward inner primer (G allele) (28 bp) 5′-CAGACTTGGTACTGAGGAAGGCGCTATG	69°C	1.0 *μ*M	4 : 1	2.5 mM	62°C
	Reverse inner primer (T allele) (23 bp) 5′-GTCTGTTTCCTTGAGCGTGCCGA	69°C	1.0 *μ*M			
	Forward outer primer (28 bp) 5′-CTGAAAACCTTTGCTGGGTCTTACATGC	69°C	0.25 *μ*M			
	Reverse outer primer (29 bp) 5′-GACCTAGTCCATCAGATGCTACCAGAGGG	69°C	0.25 *μ*M			
rs3757173	Forward inner primer (T allele) (26 bp) 5′-GACCTTATTCCCTTCCCTGAAATGAT	64°C	1.0 *μ*M	4 : 1	2.5 mM	53°C
	Reverse inner primer (C allele) (27 bp) 5′-CCTTAGCTGCAGACTAAGGTGGTATTG	64°C	1.0 *μ*M			
	Forward outer primer (28 bp) 5′-TTAAACCATTCAGTCCCCTAGAATAGCA	64°C	0.25 *μ*M			
	Reverse outer primer (28 bp) 5′-TAAAATCTTCCTACTGCCCATCTCTTTC	64°C	0.25 *μ*M			

*IRF5 *						
rs4728142	Forward inner primer (A allele) (26 bp) 5′-GTCACACCCCAAAAAGCTCTGAGACA	68°C	2.0 *μ*M	5 : 1	1.25 mM	55°C
	Reverse inner primer (G allele) (26 bp) 5′-CCTTCCTCCCCATTTCTTACTAACCCC	68°C	2.0 *μ*M			
	Forward outer primer (28 bp) 5′-GAAAGGTGGAGACTCCGAGTGTAGAGGT	68°C	0.2 *μ*M			
	Reverse outer primer (28 bp) 5′-GACAGAGCGATACTCCGTCTCAAAAGAA	68°C	0.2 *μ*M			

**Table 4:** Frequencies of alleles and genotypes for *STAT4  *rs7574865 and rs10168266, and *TNFAIP3  *rs2230926 in SLE patients and healthy control subjects of each ethnicity.

Ethnicity	Locus	Frequency		*P *value	* P *value (Bonferroni adjusted)	OR (95% CI)
		SLE patients	Healthy controls			
	*STAT4* rs7574865					
Malay		*n* = 93	*n* = 110			
	Allele					
	G^†^	104 (55.9%)	148 (67.3%)	—	—	1.00
	T	82 (44.1%)	72 (32.7%)	0.019*	0.133	1.62 (1.08–2.43)
	Genotype					
	GG^†^	29 (31.2%)	51 (46.4%)	—	—	1.00
	GT	46 (49.5%)	46 (41.8%)	0.276	NA	1.76 (0.95–3.24)
	TT	18 (19.3%)	13 (11.8%)	0.137	0.959	2.44 (1.04–5.68)
Chinese		*n* = 245	*n* = 294			
	Allele					
	G^†^	263 (53.7%)	366 (62.2%)	—	—	1.00
	T	227 (46.3%)	222 (37.8%)	0.004*	0.028*	1.42 (1.12–1.82)
	Genotype					
	GG^†^	69 (28.2%)	114 (38.8%)	—	—	1.00
	GT	125 (51.0%)	138 (46.9%)	0.345	NA	1.50 (1.02–2.20)
	TT	51 (20.8%)	42 (14.3%)	0.046*	0.322	2.01 (1.21–3.33)
Indian		*n* = 22	*n* = 26			
	Allele					
	G^†^	30 (68.2%)	30 (57.7%)	—	—	1.00
	T	14 (31.8%)	22 (42.3%)	0.290	NA	0.64 (0.27–1.47)
	Genotype					
	GG^†^	9 (40.9%)	7 (26.9%)	—	—	1.00
	GT	12 (54.5%)	16 (61.5%)	0.624	NA	0.58 (0.17–2.01)
	TT	1 (4.6%)	3 (11.6%)	0.382	NA	0.26 (0.02–3.06)

	*STAT4* rs10168266					
Malay		*n* = 93	*n* = 110			
	Allele					
	C^†^	104 (55.9%)	153 (69.5%)	—	—	1.00
	T	82 (44.1%)	67 (30.5%)	0.005*	0.035*	1.80 (1.20–2.71)
	Genotype					
	CC^†^	26 (28.0%)	53 (48.2%)	—	—	1.00
	CT	52 (55.9%)	47 (42.7%)	0.061	0.427	2.26 (1.22–4.16)
	TT	15 (16.1%)	10 (9.1%)	0.128	0.896	3.06 (1.21–7.73)
Chinese		*n* = 245	*n* = 294			
	Allele					
	C^†^	266 (54.3%)	363 (61.7%)	—	—	1.00
	T	224 (45.7%)	225 (38.3%)	0.014*	0.098	1.36 (1.07–1.73)
	Genotype					
	CC^†^	69 (28.2%)	108 (36.7%)	—	—	1.00
	CT	128 (52.2%)	147 (50.0%)	0.604	NA	1.36 (0.93–2.00)
	TT	48 (19.6%)	39 (13.3%)	0.047*	0.329	1.93 (1.15–3.24)
Indian		*n* = 22	*n* = 26			
	Allele					
	C^†^	30 (68.2%)	35 (67.3%)	—	—	1.00
	T	14 (31.8%)	17 (32.7%)	0.929	NA	0.96 (0.41–2.27)
	Genotype					
	CC^†^	9 (40.9%)	11 (42.3%)	—	—	1.00
	CT	12 (54.5%)	13 (50.0%)	0.753	NA	1.13 (0.35–3.67)
	TT	1 (4.6%)	2 (7.7%)	0.654	NA	0.61 (0.05–7.88)

	*TNFAIP3* rs2230926					
Malay		*n* = 93	*n* = 110			
	Allele					
	T^†^	181 (97.3%)	203 (92.3%)	—	—	1.00
	G	5 (2.7%)	17 (7.7%)	0.025*	0.175	0.33 (0.12–0.91)
	Genotype					
	TT^†^	88 (94.6%)	93 (84.5%)	—	—	1.00
	TG	5 (5.4%)	17 (15.5%)	0.021*	0.147	0.31 (0.11–0.88)
	GG	0 (0%)	0 (0%)	NA	NA	NA
Chinese		*n* = 245	*n* = 294			
	Allele					
	T^†^	476 (97.1%)	563 (95.7%)	—	—	1.00
	G	14 (2.9%)	25 (4.3%)	0.222	NA	0.66 (0.34–1.29)
	Genotype					
	TT^†^	231 (94.3%)	270 (91.8%)	—	—	1.00
	TG	14 (5.7%)	23 (7.8%)	0.335	NA	0.71 (0.36–1.42)
	GG	0 (0%)	1 (0.4%)	0.361	NA	NA
Indian		*n* = 22	*n* = 26			
	Allele					
	T^†^	44 (100%)	52 (100%)	—	—	1.00
	G	0 (0%)	0 (0%)	0	NA	NA
	Genotype					
	TT^†^	22 (100%)	26 (100%)	—	—	1.00
	TG	0 (0%)	0 (0%)	NA	NA	NA
	GG	0 (0%)	0 (0%)	NA	NA	NA

^†^Reference category; *P < 0.05.

**Table 5:** Association of each SNP with the Malaysian SLE, resulting from combined analysis of the three ethnicities.

Gene	SNP	Minor allele	OR (95% CI)	*P*-value (Bonferroni adjusted)
*STAT4 *	rs7574865	T	1.337 (0.948–1.885)	0.686
	rs10168266	T	1.435 (1.143–1.802)	0.014*
	rs7601754	G	0.800 (0.589–1.085)	NA

*TNFAIP3 *	rs2230926	G	0.522 (0.273–0.999)	0.350
	rs3757173	C	1.660 (1.088–2.531)	0.133

*IRF5 *	rs4728142	A	1.272 (0.934–1.733)	0.889
	rs729302	C	0.947 (0.768–1.168)	NA

*P < 0.05.

**Table 6:** Main data extracted from the studies included in the meta-analysis of *STAT4  *rs10168266.

Study	Year	Ethnicity	Minor allele	SLE patients		Controls		OR (95% CI)
				Total	MAF	Total	MAF	
Kawasaki et al. [6]	2008	Japanese	T	308	0.378	306	0.258	1.75 (1.39–2.63)
Namjou et al. [23]	2009	European	A	2583	0.251	3099	0.184	1.49 (1.35–1.65)
		Korean	A	661	0.400	781	0.289	1.64 (1.40–1.91)
Yang et al. [24]	2010	Hong Kong Chinese	T	1484	0.4543	1484	0.3369	1.64
Kim et al. [25]	2013	Korean	A	553	0.395	663	0.297	1.55 (1.31–1.83)
Current study	2013	Malay	T	93	0.441	110	0.305	1.80 (1.20–2.71)
		Malaysian Chinese	T	245	0.457	294	0.383	1.36 (1.07–1.73)
